# Fear of cancer recurrence among young breast cancer patients: a qualitative study guided by social ecological systems theory

**DOI:** 10.3389/fpsyg.2026.1905384

**Published:** 2026-08-18

**Authors:** Fei Qin, Huode Hu, Yu Zhu, Shuman Wang, Lijuan Zhang, Ziying Wang, Hongwei Wan

**Affiliations:** 1 Department of Nursing, Shanghai Proton and Heavy Ion Center, Shanghai, China; 2 Shanghai Key Laboratory of Radiation Oncology, Shanghai, China; 3 Shanghai Engineering Research Center of Proton and Heavy Ion Radiation Therapy, Shanghai, China

**Keywords:** breast cancer, fear of cancer recurrence, qualitative study, social-ecological systems theory, young patient

## Abstract

**Objective:**

To explore the experiences and needs related to fear of cancer recurrence among young breast cancer patients from the perspective of Social Ecological Systems Theory.

**Methods:**

A descriptive qualitative study design was employed. From September 1 to November 30, 2025, semi-structured interviews were conducted with 16 young breast cancer patients. Guided by social ecological systems theory (SET) as the theoretical framework, data were analyzed using content analysis.

**Results:**

A total of three themes and 10 subthemes were identified: (1) Microsystem (Individual Level): hypervigilance to physical symptoms; treatment-related sequelae and ongoing therapy; and from information seeking to fear; (2) Mesosystem (relational level): guilt and frustration arising from role reversal; communication barriers with family and friends; and ambivalent experiences within peer support networks; and (3) Macrosystem (Societal Level): medical advancement entangled with financial burden; career disruption and uncertainty about future employability; chaotic online health information; and insufficient and emerging psychological support systems.

**Conclusion:**

Fear of cancer recurrence (FCR) among young breast cancer patients is rooted not only in individual bodily vigilance and cognitive biases but also deeply embedded within broader social structures, including family relationships, peer support networks, healthcare and economic policies, occupational environments, information ecosystems, and sociocultural expectations. Therefore, effective management of FCR should extend beyond individual-level psychological interventions and adopt a comprehensive, multi-level approach that addresses the diverse contextual factors shaping patients' experiences.

## 1 Introduction

Breast cancer is the most common malignant tumor among women, and the incidence among young women has shown a year-by-year increasing trend (Guo et al., 2019). Based on the international consensus of the European School of Oncology (ESO) and the European Society for Medical Oncology (ESMO), young breast cancer patients are defined as those aged ≤ 40 years at diagnosis (Paluch-Shimon et al., 2022). In China, more than 50,000 women under 40 years of age are diagnosed with breast cancer annually, making young breast cancer a significant public health concern (Guo et al., 2019). Young breast cancer patients exhibit more aggressive clinical and biological features than their older counterparts, including higher rates of lymph node involvement, increased tumor proliferative activity, and a greater prevalence of genetic susceptibility mutations, resulting in an elevated risk of recurrence and metastasis (Margenthaler, 2025; Wang et al., 2025). Evidence suggests that younger age is consistently associated with higher levels of fear of cancer recurrence among cancer survivors (Gormley et al., 2022).

Fear of cancer recurrence (FCR) refers to the fear, worry, or concern that cancer may recur or progress and is recognized as one of the most common unmet psychological needs among cancer survivors (Lebel et al., 2016). Approximately 60.7%−70.0% of young breast cancer patients experience moderate-to-high levels of FCR, with nearly 29% continuing to report elevated FCR even 5 years after diagnosis (Gormley et al., 2022; Schapira et al., 2022). Excessive FCR is associated with substantial physical, psychological, and social consequences, including increased symptom burden, psychological distress, poorer treatment adherence, impaired quality of life, and even suicidal risk (Lebel et al., 2016; Patel et al., 2024). Therefore, fear of cancer recurrence among young breast cancer patients warrants particular attention.

Previous quantitative studies have identified a range of factors associated with FCR, including younger age, cancer stage, persistent physical symptoms, negative emotions, higher perceived stress, and maladaptive illness perceptions (Jamal et al., 2025; Li et al., 2024). Although quantitative studies can identify factors associated with fear of FCR, they provide limited insight into patients' lived experiences and the emotional processes underlying FCR. In contrast, qualitative research offers a deeper understanding of patients' perspectives, enabling a more comprehensive exploration of their thoughts, feelings, and experiences related to FCR (Su et al., 2025). Existing qualitative studies on FCR remain limited and have primarily focused on patients from Western developed countries (Chen et al., 2025; Ye et al., 2023). The experiences and support needs of patients living in different sociocultural contexts, such as China, remain insufficiently understood. China has a unique sociocultural context characterized by values such as filial piety, traditional gender roles, and fatalistic beliefs, all of which may profoundly influence young breast cancer patients' attitudes toward and responses to FCR (Liu et al., 2025). To date, qualitative research on FCR has predominantly focused on individual psychological experiences, with limited attention to the broader social and environmental contexts in which these fears develop (Mardani et al., 2025; Thewes et al., 2016). Applying Social Ecological Systems Theory may facilitate a more comprehensive understanding of how multilevel influences shape FCR among young breast cancer patients.

As a negative psychological response, FCR may have implications across multiple ecological levels, including physiological and psychological burdens at the individual level (microsystem), family, peer, and social relationships at the interpersonal level (mesosystem), and broader societal challenges related to social support systems, healthcare resources, cultural expectations, and employment environments at the societal level (macrosystem) (Xiong et al., 2025). The social-ecological systems theory (SET) emphasizes the interactions between individual development and the surrounding environment, forming a comprehensive ecological system. It categorizes the human social-ecological system into three levels: the microsystem, which comprises the individual system, including biological, psychological, and social subsystems that affect the person; the mesosystem, consisting of small-scale groups such as families and other social networks; and the macrosystem, encompassing organizations, communities, and broader sociocultural contexts (Zastrow et al., 2019). Interactions among these ecological levels play a critical role in facilitating patients' adaptation to illness. This multilevel analytical perspective provides a comprehensive framework for understanding the complexity of patients' emotional experiences and the diverse factors that shape them. Moreover, SET has been widely applied in studies examining patients' psychological outcomes and caregivers' experiences, demonstrating its value in understanding health-related psychosocial phenomena (Akyirem et al., 2025; Du et al., 2025; Jin et al., 2025). Given that FCR among young breast cancer patients is shaped by interacting influences across the microsystem, mesosystem, and macrosystem, SET provides a useful framework for understanding this phenomenon. Therefore, this study employed SET to explore the psychological experiences and support needs related to FCR among young breast cancer patients in China.

In summary, this qualitative study guided by SET is to explore the experiences and needs related to FCR among young breast cancer patients from a multilevel perspective. The findings may provide valuable insights for the development of targeted psychological and supportive care interventions for this population.

## 2 Method

### 2.1 Study design

This study adopted a descriptive qualitative design informed by Social Ecological Systems Theory (SET) to explore the experiences and support needs related to fear of cancer recurrence among young breast cancer patients. The study design and reporting adhered to the Consolidated Criteria for Reporting Qualitative Research (Tong et al., 2007).

### 2.2 Participants

Purposive sampling was used in this study (Campbell et al., 2020). Female patients admitted to the breast ward of the Shanghai Proton and Heavy Ion Hospital between September 1 and November 30, 2025, were recruited as study participants. The inclusion criteria were as follows: (a) age between 18 and 40 years; (b) scores of the Fear of Progression Questionnaire (FoP-Q-SF) ≥34; (c) adequate ability to read and communicate in Chinese; (d) a histopathological or cytological diagnosis of breast cancer (Chinese Anti-Cancer Association Breast Cancer Committee, and Oncology Society of Chinese Medical Association Breast Oncology Group 2023); and (e) voluntary participation in the study with written informed consent provided. The exclusion criteria were as follows: (a) sudden deterioration of cancer during the study period; and (b) receipt of psychological therapy during the study period.

Maximum variation sampling was applied based on participants' educational level, place of residence, occupation, number of children, and treatment regimen, with the aim of ensuring that the sample comprehensively reflected diverse patterns in the development of FCR and facilitating an in-depth exploration of the heterogeneity of FCR. Sample size was determined by data saturation. According to the recommendation of Francis et al., data saturation was considered achieved when no new information emerged in three consecutive interviews (Francis et al., 2010). In this study, redundancy in the data was observed after the analysis of interviews with 13 participants; therefore, three additional participants were recruited. As no new themes or information emerged, recruitment was discontinued. The final sample comprised 16 female participants. Interviewees were identified using numerical codes (P1–P16) to protect participants' privacy.

### 2.3 Data collection

Our research team consisted of researchers with formal training in qualitative research and backgrounds in psychology, including nursing PhD scholars, postgraduate nursing students, clinical nurses, and a psychologist. Two postgraduate members (Fei Qin and Shuman Wang) of the team developed a preliminary interview guide based on a review of domestic and international literature on FCR and guided by SET. Two breast cancer patients were recruited to participate in pilot interviews; data from these interviews were not included in the formal qualitative analysis. The research team held regular meetings to revise and refine the interview guide based on issues encountered during the pilot interviews and feedback from participants, resulting in the final version. To minimize emotional distress and avoid repeated stimulation, sensitive terminology used during the interviews was adjusted; for example, the term “the disease” was used in place of “breast cancer.” This approach aimed to create a more comfortable interview environment and encourage participants to express their experiences and feelings more openly. The final interview guide is presented in Table 1.

**Table 1:** Interview guide.

Level	No.	Interview question
Microsystem (individual level)	1	Since being diagnosed with breast cancer, have you experienced worries or fears about disease recurrence? Could you describe these experiences?
	2	When you worry about disease recurrence, what physical reactions or bodily sensations do you usually experience?
	3	When these worries arise, what thoughts, images, or emotions typically come to your mind?
	4	Have you actively searched for information about breast cancer or recurrence? How has the information you found influenced your feelings and concerns?
Mesosystem (relational level)	5	How have these worries about disease recurrence affected your family life, work, or social roles?
	6	Have you shared your concerns about recurrence with family members, friends, colleagues, or fellow patients? How did their responses affect you?
	7	During interactions with others, have you experienced feelings of support, pressure, or conflict related to these concerns?
Macrosystem (societal level)	8	How do you perceive the support provided by hospitals, communities, or society in helping you cope with these emotional concerns?
	9	Have medical information, online platforms, or media reports influenced your worries about disease recurrence? If so, in what ways?
	10	What social factors do you think influence your worries or concerns? Could you provide some examples?
Coping and support needs	11	When worries about recurrence arise, what strategies do you usually use to cope? Which strategies do you find most helpful?
	12	What types of support would you like to receive from healthcare providers, family members, or society to better manage these concerns?

Data were collected through semi-structured, face-to-face interviews. The interviewers (Fei Qin and Huode Hu) were senior female head nurses in the oncology radiotherapy department with extensive clinical experience. They had established trusting relationships with patients, were familiar with the clinical context, and had received training in qualitative research methods and fundamental interviewing skills. Interviews were conducted in a quiet and private consultation room to allow participants to freely and fully express their experiences and thoughts. Before the interviews, participants were informed of the study's purpose, methods, and significance, and were assured of the principles of non-maleficence and confidentiality. Written informed consent was obtained prior to participation. General demographic information was then collected, followed by one-on-one interviews. All interviews were audio-recorded, and non-verbal information was documented. No other individuals were present during the interview sessions. All interviews were conducted in Chinese and lasted approximately 60–90 min.

### 2.4 Data analysis

Within 24 h of each interview, audio recordings were transcribed verbatim and imported into NVivo 12 for data management and analysis. Qualitative content analysis was conducted using Social Ecological Systems Theory (SET) as the guiding framework for data interpretation and categorization, thereby ensuring a theory-informed analytical process (Lu et al., 2024). Two researchers (Fei Qin and Huode Hu) independently analyzed the data. Following repeated readings of the transcripts, meaning units were identified and coded, with the microsystem, mesosystem, and macrosystem serving as initial coding categories. Themes and subthemes were subsequently developed through iterative comparison of codes and team discussions. To enhance credibility, the findings were returned to participants for member checking. All interviews were conducted in Chinese; excerpts included in this manuscript were translated into English by Fei Qin and reviewed by Hongwei Wan.

### 2.5 Rigor

To ensure the trustworthiness of this study, multiple strategies were employed to establish credibility, dependability, confirmability, and transferability (Morse, 2015). The researchers (Fei Qin and Huode Hu) had extensive clinical experience and professional expertise in this field; however, their professional backgrounds and involvement in data analysis may have introduced potential interpretive bias. To address this concern, interview transcripts and preliminary analytical findings were promptly returned to participants for member checking, ensuring that the researchers' interpretations accurately reflected participants' lived experiences and perspectives. Throughout the analytical process, the research team held regular reflexive meetings to critically discuss and review the emerging findings. All researchers independently examined the de-identified interview transcripts to verify transcript accuracy and identify potential factors that could influence data interpretation. The subthemes and themes were collaboratively reviewed and refined by all authors through an iterative process until consensus was achieved. This iterative and collaborative process incorporated multiple perspectives into data interpretation, thereby minimizing researcher bias and strengthening the credibility and confirmability of the findings. Furthermore, detailed descriptions of the research context, participant characteristics, and data collection and analysis procedures were provided to enable readers to assess the transferability of the findings.

### 2.6 Ethics

The study was approved by the Shanghai Proton and Heavy Ion Center Institutional Review Board (No: 2308-67-02-2412A). All interviews were conducted after obtaining written informed consent from the participants, who were free to withdraw at any time. Confidentiality and anonymity were strictly maintained throughout the study.

## 3 Results

Among the 18 young breast cancer patients who met the eligibility criteria, two declined participation due to lack of interest or the need to rest for health reasons. The remaining 16 patients voluntarily participated in the study, provided written informed consent, and all completed the interviews without withdrawal. All 16 participants were female, aged 27 to 40 years. Nine were married, four were unmarried, two were divorced, and one was widowed. Fourteen participants held a bachelor's degree or higher. Disease stages included 5 at stage I, 8 at stage II, 2 at stage III, and 1 at stage IV, and all had undergone surgical treatment. The duration of cancer treatment ranged from 6 to 36 months. The demographic and clinical characteristics of the participants are presented in Table 2.

**Table 2:** Participants' characteristics (*n* = 16).

ID	Age (years)	Education	Residence	Marital status	Occupation	Number of children	Disease duration (months)	Disease stage	Previous treatment(s)
P1	40	Bachelor	Guangzhou	Widowed	Unemployed	0	16	I B	Surgery + Chemotherapy + Endocrine Therapy
P2	28	Bachelor	Jilin	Unmarried	Flight attendant	0	8	II A	Surgery + Chemotherapy + Targeted Therapy
P3	38	College	Wuhan	Married	Employed	1	9	I A	Surgery + Chemotherapy + Targeted Therapy
P4	27	Bachelor	Henan	Unmarried	Unemployed	0	9	II B	Surgery + Chemotherapy + Targeted Therapy
P5	40	Master	Shanghai	Married	Employee	1	24	IV	Chemotherapy + Targeted Therapy
P6	40	Bachelor	Shaanxi	Married	Employee	2	36	II	Surgery + Chemotherapy + Targeted + Endocrine Therapy
P7	39	Bachelor	Guangzhou	Unmarried	Unemployed	0	12	I B	Surgery + Chemotherapy + Targeted + Endocrine Therapy
P8	40	High School	Sichuan	Divorced	Freelancer	2	12	III B	Surgery + Chemotherapy + Endocrine Therapy
P9	38	Bachelor	Hainan	Married	Employee	1	9	II A	Surgery + Chemotherapy + Endocrine Therapy
P10	30	Bachelor	Guangzhou	Unmarried	Physician	0	12	II A	Surgery + Targeted + Endocrine Therapy
P11	39	Bachelor	Anhui	Married	Employee	2	7	II A	Surgery + Endocrine Therapy
P12	37	Bachelor	Suzhou	Married	Employee	2	10	III C	Surgery + Chemotherapy + Prosthesis
P13	35	Doctorate	Shanghai	Divorced	Teacher	1	6	I B	Surgery + Chemotherapy + Endocrine Therapy
P14	34	High School	Anhui	Married	Staff	2	8	I B	Surgery + Chemotherapy
P15	38	Bachelor	Suzhou	Married	Nurse	1	8	II B	Surgery + Chemotherapy + Endocrine Therapy
P16	37	Master	Shanghai	Married	Finance	1	6	II A	Surgery + Chemotherapy

**Figure 1:**
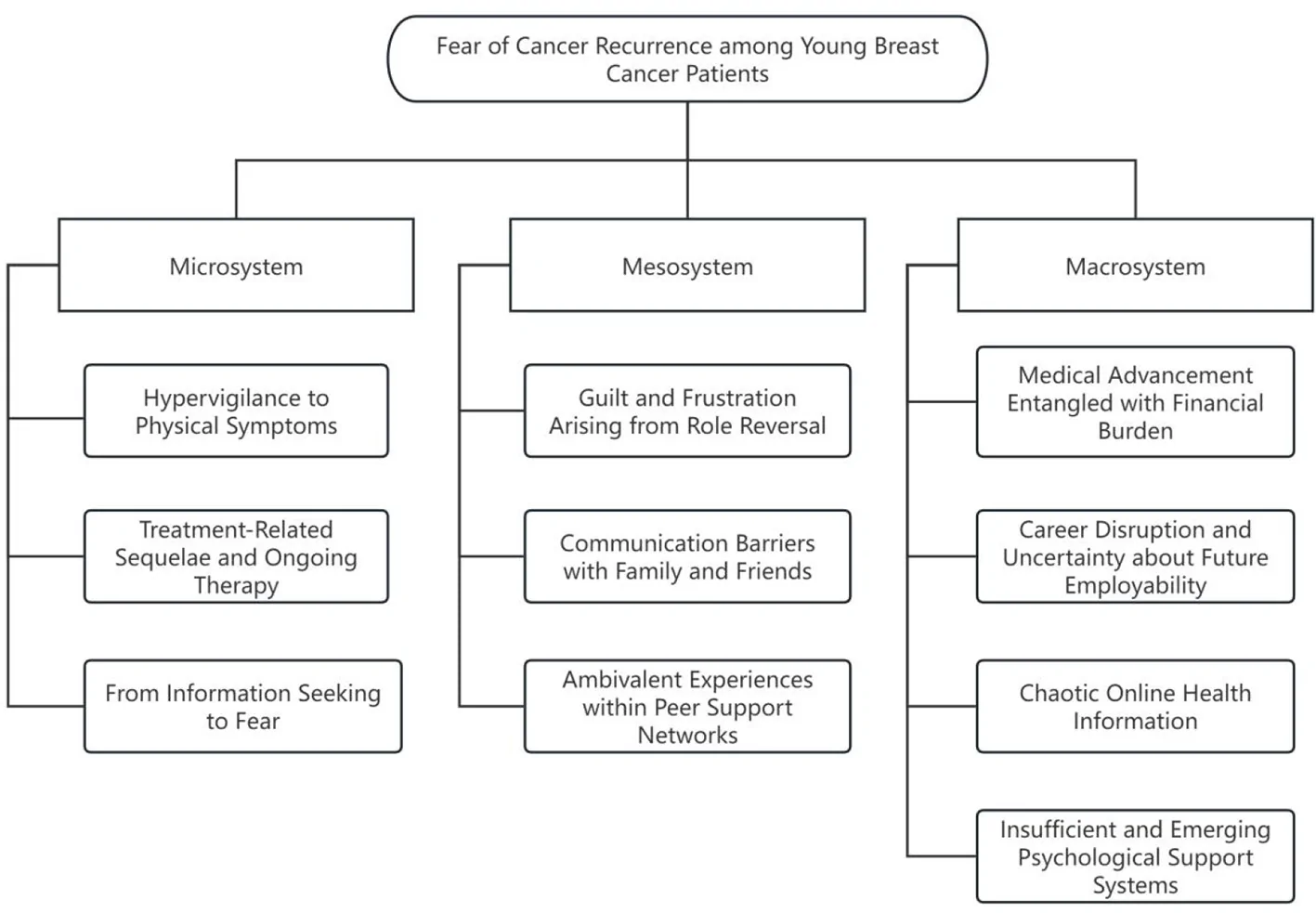
Main themes and subthemes.

Based on the SET, the experiences and needs of FCR among young breast cancer patients were categorized into three main themes and ten subthemes. Details are presented in Figure 1.

### 3.1 Theme 1: microsystem (individual-level)

#### 3.1.1 Hypervigilance to physical symptoms

Following diagnosis, young breast cancer patients reported a persistent FCR that permeated their daily lives. Ordinary bodily discomforts or minor physical changes were often immediately interpreted as signs of recurrence or metastasis, leading to heightened anxiety, fear, and emotional distress.

“*I scare myself. When my leg suddenly hurts, I think about bone metastasis. If I cough occasionally, I worry that something is wrong with my lungs. I keep thinking that this might be the end for me.”* (P1)

“*Every time I undergo medical tests, I worry about the possibility of recurrence. Before each examination, I tell myself, ‘What if this time it's not okay?”'* (P6)

“*This morning, I felt a hard lump under my arm. The doctor told me it was just a surgical scar and not a problem, but I still doubted whether the radiotherapy was sufficient or whether the surgery was complete. I kept wondering if recurrence was possible. I was truly frightened.”* (P8)

#### 3.1.2 Treatment-related sequelae and ongoing therapy

Young breast cancer patients reported that treatment-related sequelae following surgery, chemotherapy, and radiotherapy—such as lymphedema, persistent pain, and functional limitations of the limbs—contributed substantially to their FCR. Patients receiving endocrine therapy described additional symptoms, including hot flashes and sleep disturbances, with the knowledge that endocrine treatment may continue for 5–10 years. These ongoing side effects served as constant reminders of the cancer experience and reinforced concerns about disease recurrence.

“*I sleep poorly at night, have tinnitus in my right ear during the day, nerve pain, and numbness in my hands and feet. These post-chemotherapy symptoms have kept me in a very low place. I can't imagine how I would cope if it all happened again.”* (P6)

“*After surgery, the doctor told me not to bear weight on my left arm. Now I don't dare do housework—even lifting a pan scares me. I feel almost like a useless person.”* (P14)

“*Endocrine medication makes me feel overwhelmed. I have hot flashes constantly, and my clothes are soaked all day—it's really unbearable. Knowing that I still have five more years of endocrine therapy ahead of me keeps reminding me that I am a patient, and it makes me even more afraid of going through everything again.”* (P15)

#### 3.1.3 From information seeking to fear

During the course of diagnosis and treatment, young breast cancer patients actively and intensively sought health-related information, particularly regarding disease recurrence. Participants described how increased knowledge obtained through self-directed information seeking intensified fear of recurrence, with recurrence-related thoughts becoming increasingly unavoidable, while cumulative uncertainty across different stages of treatment further amplified this fear.

“*The more you know, the more afraid you become of recurrence. Once you understand all the treatment options and have seen so many cases of recurrence, it's impossible not to think about it. You always imagine worse outcomes.”* (P10)

“*I search for information myself, and I know that this disease doesn't end with just one surgery. Because recurrence is a possibility, I can't help thinking about it and feeling worried.”* (P1)

“*Having this disease feels like fighting monsters in a game. First comes the diagnosis, and you search online to figure out what it is and what to do. While waiting for pathology results, you worry about whether it's triple-negative. Then you stress over which drugs to use. At every stage, you wonder how effective the treatment will be. Now, at the final stage, I'm stuck thinking about what would happen if it recurred—whether radiotherapy would still be possible.”* (P15)

### 3.2 Theme 2: mesosystem (relational-level)

#### 3.2.1 Guilt and frustration arising from role reversal

In the Chinese cultural context, young women are often expected to assume primary caregiving roles within the family. Following a breast cancer diagnosis, patients experienced a profound role reversal, shifting from caregivers to care recipients. FCR was therefore not only self-oriented but also deeply relational, rooted in concerns about becoming a renewed emotional, practical, and financial burden on family members. This sense of guilt and frustration significantly intensified their fear.

“*I just became a mother, and I'm really afraid of recurrence. I'm afraid there will be no one to buy clothes or toys for my child, and that I won't be able to accompany them as they grow up.”* (P3)

“*I used to take care of everyone in my family, but now my role has completely changed—I'm the one being taken care of, and I feel very guilty. If recurrence or metastasis happens, going through the whole process of seeking treatment again would be extremely painful. I keep thinking about my husband, my child, my siblings, and my parents—they would suffer an even greater blow. That's very hard for me to accept.”* (P6)

“*I know my family loves me even more than I love myself. I'm not afraid of recurrence, metastasis, or even death, but I'm afraid that if recurrence happens, it will mean another huge expense and my family will have to support me financially again. I really don't know what to do.”* (P11)

#### 3.2.2 Communication barriers with family and friends

Family and friends often attempted to protect patients emotionally by encouraging optimism, minimizing negative emotions, or emphasizing future-oriented plans (e.g., retirement travel). However, these well-intentioned strategies frequently triggered fear rather than comfort. Some participants felt that their genuine fears were invalidated or unseen, leading to increased emotional loneliness and, in some cases, social withdrawal.

“*My husband tries to comfort me by saying that when he retires, he'll take me traveling. But I snap back and say, ‘I don't even know if I'll live until then.' Whenever he talks about the future, I can't help but feel afraid of death or avoid thinking about what's ahead.”* (P5)

“*Friends, colleagues, and even family members can't really feel this kind of fear I have. They always tell me to stay positive so I can recover, but their concern and visits just keep reminding me of my identity as a cancer patient. So I choose to avoid them.”* (P7)

“*When I feel scared or anxious, my family tells me not to look things up online and not to search randomly, but I just can't stop myself.”* (P9)

#### 3.2.3 Ambivalent experiences within peer support networks

Peer support groups provided young breast cancer patients with opportunities for shared understanding, mutual encouragement, and emotional resonance. However, some participants reported feelings of marginalization within these groups. Older patients often offered comfort based on completed life experiences, inadvertently highlighting young patients' fears of an interrupted or unrealized future. Additionally, hearing about recurrence, metastasis, or death among peers frequently triggered vicarious trauma, intensifying fears of “being next.”

“*I don't really like communicating with much older patients. If I were in my 50s or 60s, had a family and children, maybe I could let go of many things. But I'm still young—I'm even afraid I won't live to their age.”* (P4)

“*There are many breast cancer patients, and we share experiences and empathize with each other. But a patient I was close to developed metastasis. I couldn't help matching myself to her—same subtype, same treatment plan… and I started wondering if I would be the next one.”* (P15)

“*Since getting sick, I've joined many patient groups. Patients can openly talk about these things, and some have returned to work. But you also see that many of them are often emotionally low, and it pulls me into reflection and doubt—am I really healed? Can I really live like a normal person? Will I relapse? These questions seem to accompany life afterward.”* (P16)

### 3.3 Theme 3: macrosystem (societal-level)

#### 3.3.1 Medical advancement entangled with financial burden

Rapid advances in medical technology have significantly improved survival prospects for young breast cancer patients. However, in the Chinese healthcare context, cutting-edge treatments such as proton and heavy ion therapy, novel breast cancer–related medications, and genetic testing are largely self-funded. While these innovations offer unprecedented hope, such hope is often accompanied by substantial financial costs. As a result, patients face a persistent dilemma between the promise of medical progress and the limits of personal economic accessibility, which intensifies FCR.

“*I'm triple-negative, and there isn't a specific targeted drug for my type. I've gone through standard chemotherapy-*−*26 rounds in total. I was lucky enough to find resources and join a clinical trial for a new AstraZeneca drug from the U.S., but it's completely self-funded. Including proton and heavy ion therapy, I've already spent a huge amount of money. I'm really afraid that if recurrence or metastasis happens, will I still be able to hold on until there's a suitable new drug? And even if there is one, will I be able to afford it?”* (P6)

“*Medical technology is advancing very fast. Ten years ago, there weren't even proton and heavy ion therapy centers, and now they're quite mature—but still very expensive. I don't think the disease is untreatable; I think the problem is that I may not be able to afford the best treatments to prevent recurrence.”* (P8)

#### 3.3.2 Career disruption and uncertainty about future employability

Young breast cancer patients are often in critical stages of career development or professional stability. The onset of illness disrupts career trajectories and generates uncertainty regarding future work capacity. Participants frequently oscillated between doubts about whether they could continue working and fears that work-related stress might trigger cancer recurrence.

“*After getting sick, I feel physically and mentally unable to cope with work, and at the same time, I'm afraid that working again might bring back the struggle with the disease.”* (P3)

“*Companies in China still care a lot about whether you can be a productive ‘workhorse.' During recruitment, there's discrimination against us as cancer patients. At the same time, I worry that high-intensity work could lead to recurrence.”* (P2)

“*I keep questioning whether I'm really healed, whether I can truly work like I used to, and whether recurrence will happen.”* (P11)

#### 3.3.3 Chaotic online health information

While the internet serves as an important source of support for young breast cancer patients, it simultaneously acts as a persistent trigger for FCR. Young patients frequently searched for disease-related information online following diagnosis, but the quality and reliability of online resources varied widely. Exposure to unverified, fragmented, or alarmist content across social media platforms such as WeChat, Xiaohongshu, and Douyin often exacerbated anxiety, leaving patients struggling to obtain a clear understanding of their condition and intensifying FCR.

“*Since getting sick, I've been searching online for information about my disease. Then my phone keeps pushing all kinds of related content to me. Because I'm so focused on my illness, I hardly miss any of it, and instead, it increases my fear of recurrence.”* (P8)

“*It feels like big data is constantly monitoring me. On Xiaohongshu, I keep seeing posts about recurrence cases, which repeatedly remind me: ‘Don't forget—you might relapse.”'* (P13)

“*In patient groups, there's often a lot of health advice, especially some popularized traditional Chinese medicine ideas about ‘trigger foods' or ‘cold and hot body types.' My family and I end up believing them, and I don't dare eat things like eggs or chicken because I'm afraid they might cause recurrence.”* (P16)

#### 3.3.4 Insufficient and emerging psychological support systems

Most young breast cancer patients became aware that FCR constituted a significant psychological burden during hospitalization. However, in practice, many hesitated to seek professional psychological help due to embarrassment, stigma, or concerns about causing additional anxiety for their families. Consequently, they often choose to endure distress alone. Participants widely expressed a desire for more diverse, accessible, and normalized psychological support options in the future.

“*I know this anxiety and fear are unhealthy and need to be addressed, but if I go to see a psychologist, it would only make my family more worried. I don't know what other ways there are to relieve this fear.”* (P10)

“*I've heard that some hospitals have group psychological programs or ‘Pink Care' support groups. I hope every hospital could have something like this, or that there could be public welfare programs in society to help us deal with these negative emotions.”* (P11)

Notably, some young breast cancer patients had begun using artificial intelligence as an alternative form of emotional support.

“*I don't like communicating with people, but I use ChatGPT now and treat it completely like a psychologist. I tell it that I'm very afraid of recurrence, that I have anxiety and sleep problems, and that I want to loosen my psychological defenses. I think subconsciously I feel it's not a human, so I don't hold anything back when talking to it.”* (P2)

“*I think human professionals have limitations in terms of knowledge, and what they can provide may mainly be emotional support. But AI is becoming more and more knowledgeable—it might be able to address different psychological problems more precisely. I really look forward to the widespread application of AI in psychological support.”* (P12)

## 4 Discussion

A notable contribution of this study is the demonstration that FCR is not solely an individual cognitive-emotional phenomenon but is embedded within a broader ecological context.

### 4.1 Differential manifestations of FCR across ecological systems

At the microsystem level, our findings confirm that symptom hypervigilance and treatment-related sequelae contribute to FCR, consistent with prior evidence (Patel et al., 2024; Tran et al., 2022). A novel insight from this study is the identification of a “knowledge–fear cycle,” in which young patients' attempts to reduce uncertainty through active health information seeking may paradoxically amplify perceived threat and recurrence risk. This finding extends the cognitive processing model of FCR proposed by Fardell et al. (2016) by demonstrating how information-seeking behaviors are embedded within a broader sociocultural and digital environment, thereby contributing to the persistence of FCR. It is noteworthy that the observed “knowledge–fear cycle” may, to some extent, reflect the educational profile of our study sample. Most participants held a bachelor's degree or higher, which may have been associated with greater health literacy, stronger abilities to access and appraise health information, and a greater tendency to use information seeking as a strategy for managing illness-related uncertainty. As a result, their information-seeking behaviors were likely to be more proactive and in-depth, making this paradoxical mechanism more readily observable in the present study.

At the mesosystem level, family responsibilities and social relationships played a significant role in shaping FCR among young breast cancer patients. Consistent with traditional Chinese cultural values, young women often assume primary caregiving roles within the family and tend to prioritize family needs over their own (Wang et al., 2025). The transition from caregiver to care recipient following a cancer diagnosis not only generated frustration but also evoked a profound sense of relational guilt. Consequently, patients' fears of recurrence were often linked not only to concerns about disease progression but also to worries about becoming an emotional and financial burden on their families. Notably, social support emerged as a double-edged sword. While support from family, friends, and peers provided emotional reassurance and practical assistance (Bu et al., 2025; Han et al., 2023), some participants reported feeling misunderstood when their fears were minimized or dismissed. Our findings further suggest that peer networks may function as a double-edged source of influence, whereby age differences and variations in disease trajectories may also generate distress. Specifically, recurrence or disease deterioration among similar-aged peers heightened patients' perceptions of personal vulnerability and intensified FCR.

At the macrosystem level, financial burden, employment uncertainty, and the information environment shaped both the experiences of FCR and the support needs of young breast cancer patients. First, despite rapid advances in medical technology, the increasing availability of novel treatments may impose substantial financial burdens, representing an important source of FCR among young breast cancer patients (Zheng et al., 2023). Our findings further extend previous evidence by showing that patients' concerns were not limited to disease recurrence itself but also included uncertainty about their ability to afford future advanced therapies and medications. Second, career disruption and employment uncertainty emerged as important contributors to FCR. Previous studies have suggested that work provides individuals with a sense of normalcy, identity, and social participation; however, cancer diagnosis and treatment often compel patients to reassess work-related decisions, creating additional challenges (Ghazal et al., 2021). Consistently, our findings indicate that young breast cancer patients experience not only interruptions to career development due to treatment demands but also persistent concerns regarding workplace discrimination and the potential impact of work-related stress on recurrence risk. These findings highlight the need for greater societal attention to survivors' vocational rehabilitation needs, including employment guidance and return-to-work support.

### 4.2 Dynamic interactions across ecological systems

This study advances the understanding of FCR from the perspective of SET by demonstrating that FCR is shaped not by isolated individual factors but by dynamic interactions across the micro-, meso-, and macrosystems. Our findings suggest that financial strain, family responsibilities, peer influences, and culturally embedded role transitions collectively heighten patients' perceived vulnerability to recurrence. In the Chinese healthcare context, the financial burden of advanced treatments and employment uncertainty may transform recurrence concerns into fears of family financial hardship and disrupted life trajectories (Li et al., 2024; Mosconi et al., 2025). These macrosystem stressors are further interpreted through mesosystem processes, whereby family-oriented cultural values may transform economic concerns into guilt and fears of becoming a burden to loved ones. Meanwhile, reluctance to disclose recurrence-related fears may reinforce emotional isolation through reduced family support. This cross-level interplay highlights that effective FCR interventions should simultaneously address multiple ecological levels, including improving healthcare and information systems at the macrosystem level, strengthening family communication and social support at the mesosystem level, and enhancing adaptive coping and threat appraisal at the microsystem level.

### 4.3 The potential of artificial intelligence in FCR management

Emerging evidence suggests that ChatGPT-based counseling interventions can significantly reduce anxiety and depression among patients undergoing chemotherapy (Akdogan et al., 2025). Consistent with this evidence, several participants in the present study reported using ChatGPT as an informal source of emotional support, perceiving it as an accessible, anonymous, and nonjudgmental platform for discussing recurrence-related fears. Most participants in this study had a bachelor's degree or higher education, were urban residents, and had relatively advantaged socioeconomic backgrounds. These characteristics may have facilitated their access to and engagement with digital health resources, potentially contributing to their proactive and extensive information-seeking behaviors. Nevertheless, it must be emphasized that these tools cannot substitute for professional mental health assessment, diagnosis, or follow-up care. Potential risks include limited empathy, algorithmic bias, misleading outputs and potential misdiagnosis, data privacy vulnerabilities, and delayed help-seeking for professional services (Erdemir and Sumbas, 2026). Therefore, while AI represents a promising adjunctive tool, it must be positioned as a supplement to—not a replacement for—human professionals, and its integration into patient care must be accompanied by clear safety referral protocols, professional oversight, and rigorous safeguards.

### 4.4 Implications

The findings of this study have several important implications for clinical practice and healthcare policy. At the individual level, healthcare professionals should strengthen symptom management, health information literacy, and uncertainty management to help young breast cancer patients develop adaptive coping strategies for FCR. At the interpersonal level, interventions should incorporate family-centered communication and age-appropriate peer support to address relational guilt, emotional validation, and recurrence-related concerns. At the societal level, efforts are needed to improve access to reliable health information, vocational rehabilitation, and psychological support services. Given the growing demand for accessible and anonymous support, integrating digital health technologies with professional psychosocial care may represent a promising approach for addressing the unmet needs of young breast cancer patients and reducing the long-term burden of FCR.

### 4.5 Strengths and limitations

This study provides valuable insights into the experiences and support needs related to FCR among young breast cancer patients. Guided by Social Ecological Systems Theory, it offers a comprehensive understanding of FCR across individual, interpersonal, and societal levels. Methodological rigor was enhanced through expert-reviewed interview guides, pilot interviews, data saturation-based sampling, and timely transcription, supporting the credibility and trustworthiness of the findings.

However, several limitations should be noted. Participants were recruited from a single radiotherapy-focused hospital, limiting generalizability. Participants were recruited through purposive sampling from a single proton and heavy ion treatment center, which may limit the transferability of the findings. In addition, most participants were highly educated, urban residents with access to advanced cancer treatment, and therefore may not represent the broader population of young breast cancer patients. These characteristics may have influenced perceptions of financial burden, healthcare accessibility, and information-seeking behaviors. Finally, although measures were taken to enhance rigor, interviewer-related bias cannot be completely excluded. Future studies should include participants from diverse healthcare settings and socioeconomic backgrounds to improve the transferability of the findings.

## 5 Conclusion

This study extends the current literature, which has predominantly focused on individual or disease-related determinants of FCR, by highlighting the multilevel and interconnected nature of recurrence-related fears. The application of a social ecological perspective provides a more comprehensive framework for understanding the unique psychological experiences of young breast cancer patients and may inform the development of multilevel interventions to address FCR.

## Data Availability

The raw data supporting the conclusions of this article will be made available by the authors, without undue reservation.
